# Autoinflammatory Features in Gouty Arthritis

**DOI:** 10.3390/jcm10091880

**Published:** 2021-04-26

**Authors:** Paola Galozzi, Sara Bindoli, Andrea Doria, Francesca Oliviero, Paolo Sfriso

**Affiliations:** Rheumatology Unit, Department of Medicine—DIMED, University of Padova, 35128 Padova, Italy; paola.galozzi@unipd.it (P.G.); sara.bindoli@phd.unipd.it (S.B.); adoria@unipd.it (A.D.); Francesca.oliviero@unipd.it (F.O.)

**Keywords:** gout, autoinflammation, therapy, IL-1 inhibitors

## Abstract

In the panorama of inflammatory arthritis, gout is the most common and studied disease. It is known that hyperuricemia and monosodium urate (MSU) crystal-induced inflammation provoke crystal deposits in joints. However, since hyperuricemia alone is not sufficient to develop gout, molecular-genetic contributions are necessary to better clinically frame the disease. Herein, we review the autoinflammatory features of gout, from clinical challenges and differential diagnosis, to the autoinflammatory mechanisms, providing also emerging therapeutic options available for targeting the main inflammatory pathways involved in gout pathogenesis. This has important implication as treating the autoinflammatory aspects and not only the dysmetabolic side of gout may provide an effective and safer alternative for patients even in the prevention of possible gouty attacks.

## 1. Introduction

The concept of autoinflammation resulted from the acknowledgment of monogenic diseases with seemingly unprovoked inflammation and without the high-titer autoantibodies or antigen-specific T cells seen in classic autoimmune diseases [1]. However, autoinflammation and autoimmunity are not sharply defined, as many diseases display features common to both conditions. This led to the concept of the immunological disease continuum, in which intermediate place was taken by polygenic diseases with prominent autoinflammatory and/or autoimmune components [2]. Gout is thus a multifactorial autoinflammatory disease.

Gout is the most common inflammatory arthritis with about 2–4% of prevalence worldwide, mainly in men over 40 and particularly in those with underlying comorbidities such as obesity, hypertension, coronary artery disease, diabetes, or metabolic diseases. The characteristic gouty flare has a distinctive clinical feature, achieving an acute painful synovitis caused by monosodium urate (MSU) crystals deposition in joints [3].

There has been an increasing amount of evidence about the autoinflammatory nature of gout. Similarly to autoinflammatory diseases, there is a malfunction of the innate immune system in gout. Indeed, hyperuricemia solely is not sufficient to induce gout; this strongly suggests further inflammatory and genetically determined elements contributing to the disease [4]. Further autoinflammatory aspects of gout are the typically self-limiting nature of acute flares and the central role of inflammatory cytokines, such as interleukin (IL)-1β, suggesting that pro- and anti-inflammatory regulatory pathways are involved in gout [5]. Recent and already consolidated autoinflammatory aspects of gout were reviewed in this work to provide important implications for treating challenging gouty inflammation.

## 2. Clinical Challenges and Differential Diagnosis

It is widely known that gout typically presents with an acute painful flare that can resolve spontaneously within a few days, with asymptomatic periods between attacks. It usually affects the first metatarsophalangeal joint, but large joints such as knee, wrist, and ankle may be involved as well, leading to a systemic acute inflammation [6].

Fever and fatigue are not uncommon symptoms during a gout attack, but have to be considered in the differential diagnostic process to infectious arthritis or, more severely, a systemic sepsis. Fever is also a prominent sign in many autoinflammatory diseases even though the fever patterns vary considerably (from episodic to continuous fever) [1]. In gout, fever can be present mostly when there is a polyarticular involvement, since the final production of IL-1β can be a possible trigger for fever in patients affected by crystal arthropathies. Although fever may be more prevalent in the case of calcium pyrophosphate crystal-induced arthritis than it is in gout, febrile systemic inflammatory diseases particularly in elderly people may be often caused by crystal-induced arthritis [7]. In general, the prevalence of fever in gout is driven by specific pyrogens (IL-1, IL-6, tumor necrosis factor (TNF)-α) with the inflammasome as a pivotal activator of the inflammatory cascade.

Cellulitis, a potentially serious skin infection caused by different types of bacteria (*β-hemolytic streptococci*, and generally group A streptococcus, i.e., *Streptococcus pyogenes*, followed by methicillin-sensitive *Staphylococcus aureus* [8]) may be clinically similar to a gouty attack, especially when involving lower limbs with concomitant redness and soft tissue swelling. In addition, it was observed that in patients with chronic gout, the polyarticular repeated attacks may induce a systemic inflammatory response syndrome (SIRS) without associated infections [9]. In addition, the uncommon axial involvement in polyarticular gout can induce a SIRS-like reaction mimicking a sepsis with the presence of a chronic crystal arthropathy [10].

Overall, distinguishing between an infection and an acute arthritis (septic or crystal-induced, like gouty arthritis) may be quite challenging. Ultrasound scans of the joints involved together with synovial fluid analysis remain the gold standard exams for the appropriate diagnosis; however, laboratory tests, including urate serum, inflammatory markers, and procalcitonin levels, and a primary immunological assessment (protein profile, immunoglobulins, etc.) should be performed to provide a global view of the patient.

## 3. Molecular Mechanisms of Gouty Inflammation

**Figure 1:**
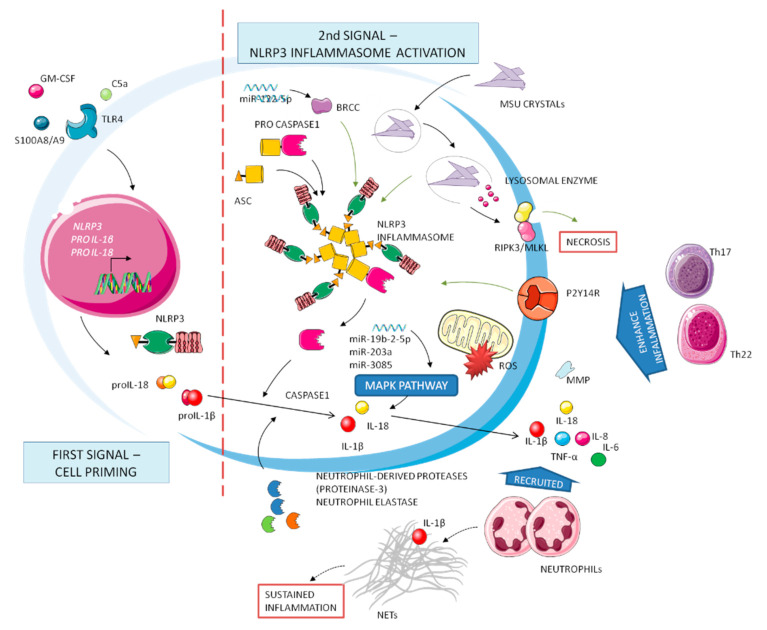
Complex network of molecular mechanisms implicated in gout. Inflammation has been defined by two stages: first signal (**left**) and second signal (**right**). Cell priming production of precursors of cytokines and inactive inflammasome molecules needs the subsequent activation step after Signal 2. IL-1β is critical to the upregulation of inflammatory processes.

In gouty inflammation, different mediators are involved with distinct effects on the initiation, amplification, attenuation, and extinction of the acute flares (Figure 1). The core event in gouty inflammation remains the activation of leukocytes by MSU crystals, danger signals leading to the initiation of the inflammatory cascade [11]. The crystals are, indeed, the first endogenous activators of NLRP3 inflammasome, a large multiprotein complex implicated in the processing of IL-1β and IL-18 precursors into their active forms.

Inflammation in gout can be illustrated as a two-phase process, requiring separate and interacting signals [12]. Cell surface receptors such as Toll-like receptors (TLR) mediate the first signal, which provides upregulated expression of inflammasome components and of IL-1β and IL-18 precursors. In the context of gout, several endogenous molecules have been proposed to act as priming signals, including the complement protein C5a, the granulocyte-macrophage colony-stimulating factor GM-CSF, and the ligands of *TLR4* receptor S100A8/A9 [13]. Exogenous, dietary-induced first signal activators include long-chain saturated fatty acids such as palmitate. The synergy between long-chain free fatty acids, released after food intake, and MSU crystals for the release of IL-1β and induction of inflammation might represent the missing link between metabolic changes, inflammasome activation, and gout attacks [14].

This priming phase is necessary but cannot trigger the inflammasome assembly and activation without the contribution of a second, more specific, and MSU crystals-mediated phase.

The oligomerization of the NLRP3 inflammasome results in the recruitment of the adapter protein ASC and auto-activation of caspase-1, that catalyze in turns the cleavage of IL-1β and IL-18 precursors into the mature forms [15]. Then, IL-1β and IL-18 are secreted from the cells via secretory lysosomes or exosomes or via the gasdermin D channel. After neutrophils recruitment, a positive loop of inflammation can continue.

During a gouty flare, MSU crystals phagocytosis induces degranulation, lysis of lysosomal and cell membranes, further recruitment of leukocytes, and release of inflammatory mediators; all of these processes contribute to the ongoing inflammation [16]. It has been recently observed that this process, notably known as pyroptosis, can be regulated by the P2Y14 receptor, linking intracellular cAMP and the gouty inflammatory cascade [17].

Neutrophils are recruited to the inflamed tissues by chemokines, such as MCP-1 and CXCL8/IL-8, and released cytokines, such as IL-1, IL-6, and TNF-α, as well as other mediators such as matrix metalloproteinases (MMP), prostaglandins, leukotrienes, ROS, and various lysosomal enzymes [18].

Inflammasome activation is surely an important, and possibly indispensable, pathway to induce inflammatory reactions in the joints. An inflammasome-independent mechanism can also activate IL-1β in gout. Neutrophil-derived proteases (proteinase-3) or elastase can indeed process the IL-1β precursor into its active form [19].

Recently, MSU crystals have been reported to be implicated in cell necrosis, mediated by the receptor-interacting protein (RIP) kinase-1, -3 and the pseudokinase mixed-lineage kinase domain-like (MLKL)-driven necroptosis pathways [20]. The complex RIPK3/MLKL can disrupt both plasma and mitochondrial membranes, leading to cell death.

MSU crystals are further implicated in the promotion of miRNAs, short non coding RNA molecules that can regulate gene expression subtly and with complexity. There are currently different miRNAs that have been found to play an activation role in acute gouty inflammation. miR-122-5p is reported to upregulate BRCC protein expression, activating the NLRP3 inflammasome [21]. Upregulation of miR-328-3p, miR-375-5p, and miR-299a positive regulates the apoptotic process by the p53 signaling pathway. Moreover, miR-203a, miR-3085, and miR-19b-2-5p regulate the MAPK signaling pathway to indirectly mediate the inflammatory response in gout [22].

While the activation of IL-1β and the role of NLRP3 in gout have been relatively well-established, the upstream pathways involved in MSU-triggered NLRP3 activation are not yet fully understood.

Considering the critical role of T cell subsets in modulating immune function, the relationship between T cell subsets and the underlying mechanisms of gouty arthritis has been increasingly considered. Enhanced immune responses mediated by Th1, Th17, or Th22 may bear a significant role in causing pro-inflammatory attacks during the development of gouty arthritis [23]. In contrast, regulatory T cell subsets such as Tregs and Th2 may inhibit the progression of gouty inflammation, carrying out an anti-inflammatory response.

Undoubtedly, IL-1β plays a pivotal role in gout; however, increasing evidence suggests other IL-1 family members can be involved in gout. IL-1α may be implicated in the local induction and amplification of gouty arthritis. IL-33, IL-37, and IL-38 have an inhibitory function in MSU crystal-induced inflammation. Furthermore, IL-37 regulates uric acid metabolism by affecting the protein level of PDZK1, a cytoskeletal controller of uric acid transport [24,25].

The importance of aberrant innate immune responses in the pathophysiology of gout is further supported by critical observations in over a decade of translational studies [26].

## 4. Resolution of Gouty Inflammation

**Figure 2:**
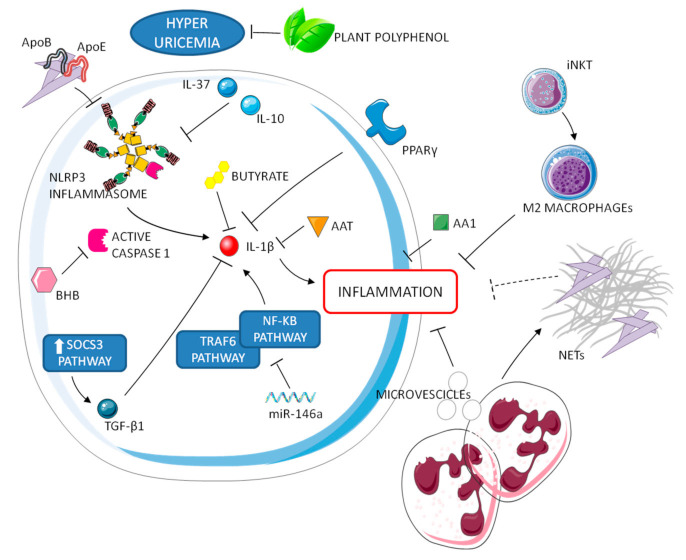
Resolution processes of gouty inflammation. Negative regulators of inflammasome and IL-1 operate in synergy with neutrophils and M2 macrophages to attenuate the inflammatory cascade.

After the protraction of the inflammatory cascade, a regulatory anti-inflammatory process attenuates gouty inflammation. It is indeed widely known that MSU-induced inflammation is characterized by spontaneous resolution [3]. Patients experiencing an acute attack improve within a few days and become chronic only if untreated. Masking MSU crystals and limiting the urate availability in circulation can help to remove the stimulatory trigger of a gout attack [27]. Known mechanisms associated with the resolution of gouty arthritis involve negative regulators of inflammasome and TLR signaling, regulators of pro-inflammatory cytokines, neutrophils, net-like structures, and pre-resolving mediators (Figure 2) [28]. Upon activation, macrophages up-regulate intracellular regulatory pathways, such as the SOCS3 pathway, believed to control the production of pro-inflammatory cytokines and for starting the production of anti-inflammatory cytokine (TGF-β1) and the secretion of soluble TNF-α receptors. TGF-β1 also reinforces the shutdown of inflammatory functions in macrophages and neutrophils, including inhibition of amplification of IL-1β signaling and downregulation of IL-1R expression [29,30].

Other anti-inflammatory cytokines (e.g., IL-10 and IL-37) have a key role in the resolution phase. IL-37, in particular, suppresses multiple innate inflammatory responses in vitro and in vivo, acting partially via inhibition of the NLRP3 inflammasome [27]. Other endogenous molecules involved in the disease self-limitation are lipoproteins ApoE and ApoB, a hormone receptor perixosome proliferator-activated receptor y (PPARy), a ketone body b-hydroxybutyrate (BHB), and an inhibitor of serine proteases α1-anti trypsin (AAT). Concerning lipoproteins, it has been reported that changes in the lipoproteins coating MSU crystals and their concentration in synovial fluid play an integral role in the self-limiting nature of an acute attack [31,32]. Both PPARy and BHB have been reported to reduce or inhibit the production of IL-1β [33,34]. It was also shown in a murine model that AAT blocked IL-1β production after MSU stimulation. Interestingly, AAT concentration and IL-1β production are linked to seasonality, since low AAT and high IL-1β levels are observed during gouty peaks in the spring and summer. Moreover, recent data demonstrate a rhythmic regulation of NLRP3 inflammasome expression and activation, linking the circadian clock to inflammatory resolution [35].

Other molecules might also contribute to the prompt resolution of inflammation in gout. The protein annexin A1 (AA1), a potential inhibitor of phospholipase A2, can decrease inflammation, thus promoting resolution in mouse models of gout [36]. In addition, miRNA 146a suppresses gouty inflammation via the downregulation of IL-1β, TNF, and NLRP3 levels by targeting TRAF6 and NF-kB signaling pathways [37]. Furthermore, exogenous substances, introduced with diet, can be involved in resolution of crystal-induced inflammation [38]. They can have immune, inflammatory, or regulatory properties. Among them, plant polyphenols are known to prevent hyperuricemia while short-chain fatty acids, such as butyrate, can suppress MSU-induced IL-1β production [39].

An interesting mechanism of auto-regulation in gout, which is also associated to autoinflammatory syndromes self-resolution, is NETosis. MSU crystals are known to induce neutrophil extracellular traps (NETs), consisting of decondensed nuclear DNA coated with cell granule enzymes released to the extracellular space [40]. This process has been shown to be dependent, at least in part, on IL-1β [40] and independent from ROS [41]. NETs have been shown to have both inflammatory and anti-inflammatory effects. While NETosis has been supposed to facilitate crystal sequestration in aggregates within tissues, limiting the inflammatory response [42], these structures have also been associated to the formation of tophi and, consequently, to the chronic evolution of the disease [43]. Interestingly, Apostolidou et al. suggested that the inflammatory attacks of familial Mediterranean fever (FMF) can be regulated by NETs through the release of IL-1β. According to their study, in fact, neutrophils from FMF patients release NETs decorated with IL-1β during disease attacks but were resistant to the release of NETs under inflammatory stimuli during remission [44]. These observations might support a dual role for NET in crystal-induced IL-1ß production and, therefore, represents an interesting issue for future studies.

Neutrophils can further release phosphatidylserine positive microvescicles that suppress inflammasome activation and consequently inhibit IL-1β release in C5a primed macrophages [45].

A recent study supports the idea that T cells, specifically type 1 NKT cells or invariant NKT (iNKT) cells, can suppress the severity of gouty inflammation, promoting M2 polarization and thus contributing to immune homeostasis [46]. This data is consistent with our observation that macrophages polarization can address the ability of the macrophages to give an inflammatory (M1-related) or non-inflammatory (M2-related) response to pathogenic crystals [47], sustaining the role of a non-inflammatory phagocytosis of the crystals in the resolution of the process as already demonstrated by our group [48].

## 5. Genetics of Gout

The familial and hereditary nature of gout has long been recognized. However, it was only in the past decade that several genes involved in rare metabolic and kidney diseases were identified as being associated with the pathogenesis of gout. Many of the identified loci include genes encoding for urate transporter, and for urate metabolism [49]. Among these, solute carrier family 2 (*SLC2A9*) and ATP-binding cassette superfamily G member 2 (*ABCG2*) have multiple variants associated with serum urate levels and, overall, the increased risk of gout. Moreover, *ABCG2* has an established key role in the onset and in severity of gout [50].

**Figure 3:**
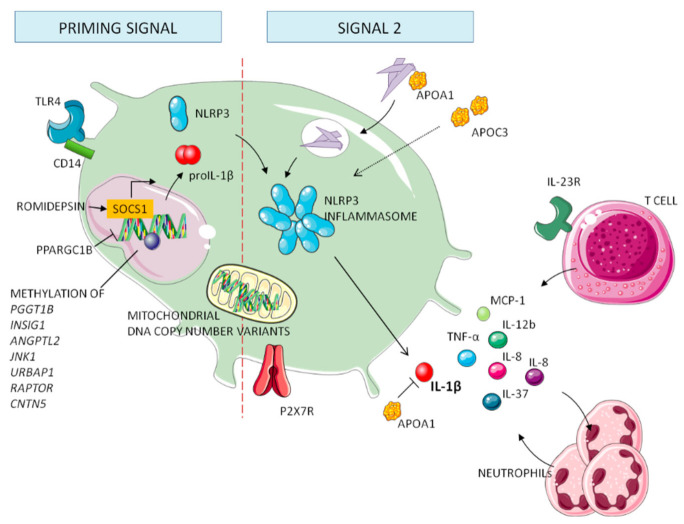
Genes involved in initiating the inflammatory response to MSU crystals. Many loci code for proteins involved in the inflammasome pathway; however, some mitochondrial and epigenetics factors have been reported to be associated with the inflammatory regulation of gouty arthritis.

In the last decade, advances in genotyping technologies have facilitated the identification of genes involved in initiating the inflammatory response to MSU crystals (Figure 3). These genetic associations yield additional findings on inflammatory regulation and shared pathways in the pathogenesis of gout. Furthermore, investigation on genes involved in autoinflammatory diseases, such as the *MEFV* gene of Familial Mediterranean fever, has obtained heterogeneous results of association with gouty inflammation [51,52].

### 5.1. Genes Involved in Processing NLRP3 Inflammasome

Many loci associated with gout are known to code for proteins directly involved in processing NLRP3 inflammasome, including membrane bound receptors, transcriptional regulators, ion channels, lipoproteins, and the inflammasome molecules (i.e., *APOA1*, *APOC3*, *CARD8*, *CD14*, *NLRP3*, *PPARGC1B*, *P2RX7*, and *TLR4*).

The *TLR4* gene, coding for a transmembrane pattern recognition receptor, an important mediator of gouty inflammation, is highly polymorphic. rs2149356 is the only variant currently associated with increased risk of gout in Han Chinese and European populations and may play a regulatory role of *TLR4* expression and IL-1 serum levels during flares [53]. These polymorphisms might affect the priming phase of the inflammatory process or might have a wider impact on the inflammatory response in these patients. The SNP rs25569190 in the *CD14* gene is reported to confer a gain-of-function to *CD14*, a co-receptor for the TLR2/4 receptor, possibly implicated in vitro in downstream inflammatory cytokine production [54]. A recent study, however, suggested an opposite role for *CD14* in self-limiting gout flares [55]. Various genetic variations in the *P2RX7* gene, coding for the P2X7 receptor implicated in inflammasome activation and probably a key regulator of IL-1β production by MSU crystals during acute gout flares, have been reported to be associated with gout: rs1653624, rs7958316, rs17525809, and rs3751142 [56]. Associated to the inflammatory signaling is also the *PPARGC1B* gene, encoding peroxisome proliferator-activated receptor γ (PPARγ) co-activator 1β. A linkage between gout incidence and polymorphisms has been reported in *PPARGC1B*, which increased NLRP3 and IL-1β expression [57]. Three SNPs were associated with gout: rs10491360, rs45520937, and rs7712296. Since PPARGC1B is known to regulate metabolism, these genetic variants might link metabolic deregulation with gouty inflammation.

Since lipoproteins can elicit inflammasome activation [31], genetic associations have been researched. rs670 in the *APOA1* gene increases the risk of gout and supports the APOA1 involvement in gouty inflammatory pathways [58]. APOA1 can bind MSU crystals and/or inhibit IL-1β production, having thus a role in initiation and/or resolution of gout attacks. The *APOC3* (rs5128) gene has a causal role in gout, decreasing the risk of gout and increasing expression of APOC3 [58]. Zewinger and colleagues, indeed, identified APOC3, a key player in triglyceride-rich lipoprotein metabolism, as a novel NLRP3 activator that promotes sterile inflammation and organ damage [59].

Along with its pathogenic role as a molecular mediator of inflammation, NLRP3’s role is further established by its genetic association with gout. rs3806268 and rs10754558 variants were associated with increased risk of gout in Chinese cohorts [60]. The rs10754558 risk allele, associated with increased expression of NLRP3 during flares, may influence the regulation of NLRP3 expression. Functional variant rs2043211 in the gene encoding caspase recruitment domain-containing protein 8 (CARD8) demonstrated an association with gout in European and Chinese cohorts [61]. Since CARD8 negatively regulates the NLRP3 inflammasome, its genetic variant might raise inflammasome activity and contribute to the sustained NLRP3 engagement in gouty episodes.

### 5.2. Genes Involved in the Downstream Cascade of NLRP3 Inflammasome

Inflammatory cytokines and cytokine receptors, downstream products of the gouty inflammatory cascade, have also been studied for genetic association or susceptibility.

The first inflammatory modulating gene associated with gout was TNF-α in a Taiwanese cohort of patients [62]. TNF-α is a well-known proinflammatory cytokine with a major role in the pathogenesis of several diseases, including gout. The rs1800630 SNP was significantly associated with augmented risk of gout. rs114362 in the *IL-1B* gene, interacting with a *CARD8* variant (rs2043211), correlates with increased expression of IL-1β, IL-6, and gout risk [63]. This reinforces the central role of IL-1β in gouty inflammation. rs4073 in the *IL-8* gene and rs7517847 in the *IL-23* receptor gene conferred increased susceptibility to gout risk [64,65]. Klück V and colleagues recently provided genetic, mechanistic, and translational evidence that the anti-inflammatory cytokine IL-37 is implicated in the pathogenesis of gout [66]. IL-12b and MCP-1, two chemokines involved in the initiation and amplification of acute flares, presented, respectively, rs3212227 and rs1024611 variants associated with increased risk for the development of gout [64].

### 5.3. Mitochondrial and Epigenetic Factors in Gout

It has been proposed that mitochondrial function and epigenetics may be associated with gout, opening up another, mainly unexplored, source of genetic contributions to inflammation in gout. Mitochondrial DNA copy number variation was consistent with emerging research showing that mitochondria are important for the colocalization of the NLRP3 and ASC inflammasome subunits, a process essential for the generation of interleukin-1β in gout [67].

A recent promoter-wide methylation study evidenced aberrant methylation changes of *PGGT1B*, *INSIG1*, *ANGPTL2*, *JNK1*, *UBAP1*, *RAPTOR*, and *CNTN5* associated to gouty inflammation [68]. Epigenetic modifiers appear also to be linked to the MSU-induced inflammatory response in gout. Cleophas and colleagues showed that romidepsin, a histone deacetylase (HDAC) 1/2 inhibitor, controlled inflammation by increasing the expression of SOCS1 and decreasing cytokines production in response to MSU crystal stimulation [69].

## 6. Therapeutic Approaches

**Figure 4:**
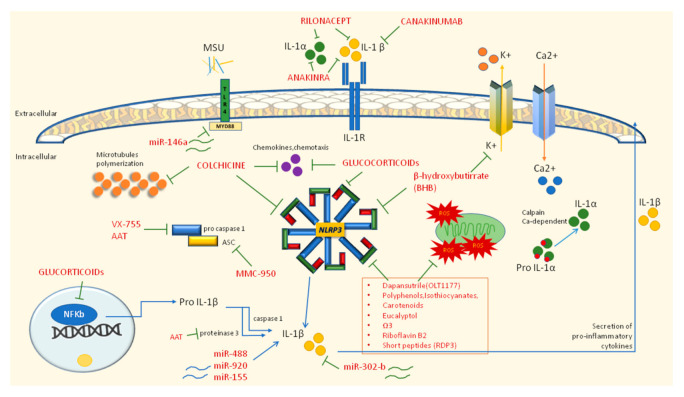
Inhibitory drugs (in **red**) of autoinflammatory mechanisms in gouty inflammation. Blue arrows indicate mechanisms of activation (i.e., maturation of IL-1α and IL-1β from precursors or the ability of miR-488 and miR-920 to induce the production of IL-1β). Green connectors identify inhibitory mechanisms towards NLRP3 or IL-1 processing.

Gout pharmacological approaches are based both on the treatment of acute flares to control the hyperinflammation status and on the prevention of attacks using urate-lowering therapies such as xanthine oxidase inhibitors (allopurinol and febuxostat), uricosurics (probenecid, benzbromarone), and URAT1 inhibitors (lesinurad) [6]. Of course, when hyperuricemia occurs in a gouty patient, low serum urate maintenance is crucial to the avoidance of other acute attacks. Gout is considered not only a dysmetabolic disorder, but is classified as an inflammatory disease, for which the activation of the innate immune system, in particular the NLRP3 inflammasome pathway, plays a central role in its pathogenesis. For this reason, targeting inflammasome and IL-1β has become crucial in treating the inflammatory component of gout (Figure 4).

### 6.1. First Line Therapy: NSAIDs, Colchicine, and Glucocorticoids

It is broadly recognized that therapy with monoclonal antibodies represents a second line choice when classical approaches are insufficient or contraindicated. The first line therapy for gouty attacks is represented by anti-inflammatory drugs (NSAIDs), colchicine and glucocorticoids, drugs recommended by ACR and EULAR guidelines as the primary approach [6,70]. Colchicine was the first drug approved for gout more than a decade ago by the FDA, and EULAR recommends it at the loading dose of 1 mg followed 1 h later by 0.5 mg on day one; the association with NSAIDs or glucocorticoids may curb the inflammatory status; however, it is mandatory to consider possible renal impairment, other drug interactions, and relevant comorbidities such as CVDs. In addition, low-dose colchicine or NSAIDs can be used in prophylaxis for at least 6 months or until 3 months after achieving the correct serum urate target. NSAIDs and glucocorticoids can be used as colchicine alternatives; however, particular attention should be paid in elderly people or those with multiple comorbidities, especially CVD and gastrointestinal bleeding for NSAIDs and hypertension and diabetes for glucocorticoids [6]. Colchicine is involved in the inflammasome-related anti-inflammatory mechanism. Indeed, colchicine acts on microtubule polymerization by binding both α- and β-tubulin to create a tubulin–colchicine complex that prevents the formation of microtubules in neutrophils and immune cells and in this way interfers with neutrophil adhesion and recruitment to inflamed tissues; moreover, the microtubule-disrupting effect hampers NLRP3 assembly and the subsequent release of IL-1β and oxygen-reactive species (ROS). In addition, the disarrangement of the microtubule structure may interfere with TNF-α release, with mast cell degranulation, and can reduce the discharge of other chemo-attractant mediators of the inflammatory response such as leukotriene B4 (LTB4) [71].

### 6.2. Second Line Therapy: IL-1 Inhibitors

The second line therapy provides for the use of IL-1 inhibitors and may be administered when patients are intolerant or refractory to traditional drugs (Table 1). To date, they include direct inhibitors of IL-1β (canakinumab and gevokizumab), selective inhibitor of the IL-1 receptor (anakinra), and a dimeric trap fusion protein (rilonacept) [7]. The efficacy of anakinra in gout was established in 2007 and despite there being no available randomized controlled trials (RCTs) to confirm the data, the drug seems to be effective in gouty patients. In particular, anakinra is adequate in patients with acute gouty arthritis unresponsive to the standard therapy and with a contraindication for NSAIDs, glucocorticoids, or colchicine [72,73]. The efficacy of rilonacept in gout has been investigated in one phase 3 RCT and in three RCTs in the prevention of flares during urate-lowering therapy [74]. The studies confirmed the efficacy of IL-1 inhibition in pain improvement and in a decrease of inflammation markers. Nevertheless, rilonacept is not currently approved by EMA nor FDA for gout. Canakinumab instead, was approved by EMA in 2013 for the treatment of gouty arthritis. The efficacy was investigated in RCTs [75], which showed a significant recovery in pain, swelling, and flare recurrence compared to that of patients taking only glucocorticoids. However, adverse events due to therapy should be considered, especially those related to infections of the upper respiratory tract, abscesses, and gastrointestinal disorders.

### 6.3. Novel Therapies Modulating Inflammatory Pathways

Recently, new treatments have been proposed to modulate and block the inflammatory pathways involved in gout pathogenesis (Table 1). Apart from colchicine, whose inhibition mechanism on NLRP3 has been aforementioned, other molecules able to hamper NLRP3 assembly should be considered. For example, beta-hydroxybutyrate, a ketone body produced in response to starvation, suppresses the potassium effluvium upstream of NLRP3, affecting the inflammasome assembly [81]; similarly, MMC-950 (also known as CP-456,773 or CRID3), a diarysolfonylurea-compound, can inhibit the NALP3-ASC oligomerization without affecting other inflammasome types [12]. Other inhibitors of inflammasome components include VX-765, also known as belnacasan, and α1 anti-trypsin (AAT), which are known to block Caspase I [78,79]. Dapansutrile, a novel β-sulfonyl nitrile compound, is an orally active small molecule that selectively inhibits NLRP3 in neutrophils and human monocyte-derived macrophages. An open-label phase IIa clinical trial (EU Clinical Trials Register, EudraCT 2016-000943-14) proved the efficacy of this molecule in reducing joint pain of gouty subjects and was well tolerated in terms of safety [80]. Another recent study [89] proved that beta-carotene (provitamin A) suppresses the NLRP3 inflammasome activation induced by MSU crystals in a mouse model. Indeed, molecular modeling and mutation assays revealed the interaction between β-carotene and the NLRP3 PYD; the oral administration of β-carotene in mice was proven to reduce the inflammation and to diminish IL-1β secretion from human synovial fluid cells isolated from gouty patients, demonstrating its inhibitory efficacy in human gout [82]. Procyanidin B2 (PCB2), a phenolic compound naturally present in grape seeds, apples, berry fruits, and tea [80], and eucalyptol [84] are known to have anti-inflammatory and antioxidant properties by suppressing NLRP3 activation in MSU-injected mice; other NLRP3 inhibitors with antioxidant properties include polydatin and resveratrol [90]; curcumin [83]; epigallocatechin gallate [12]; riboflavin (vitamin B2) [85]; and Omega-3 fatty acids (u-3 FAs) [86]. In addition, other new natural peptides are emerging as possible anti-gout treatments such as rice-derived-peptide-3 (RDP3), obtained from the water extract of shelled Oryza sativa fruits in China [87].

Many other flavonoids are reported to exert anti-inflammatory effects on mouse models of gouty arthritis, inhibiting both stages of the NLRP3 inflammatory process. Overall, polyphenols (i.e., flavonoids, stilbenoids, and phenols), triterpenoids, isothiocyanates, and carotenoids play a pivotal role in many inflammatory conditions including gouty arthritis; therefore, different phytochemicals could represent a suitable pharmacological approach or, at least, a complementary treatment in addition to the standard therapy for the management of persistent inflammatory gout [88]. Finally, in recent years, expanding evidence has pointed out that long-noncoding RNAs (lncRNAs) and micro-RNAs (miRNAs) may be specifically expressed and involved in the regulation of inflammatory gouty arthritis. Studies from murine models observed that in miR-146a knockout mice, TNF receptor associated factor 6 (TRAF) and interleukin-1 receptor associated kinase (IRAK1) were upregulated; thus, it was supposed that miR-146a can downregulate the levels of pro-inflammatory cytokines in gout. Similarly, miR-302b is involved in a downregulatory pathway, while miR-155, miR-488 and miR-920 are known to induce the production of pro-inflammatory cytokines. Among lncRNAs, ANRIL upregulates NLRP3. Overall, lncRNAs and miRNAs, may function as regulators of the pathological processes of gout and might be used for diagnosis but also as therapeutic targeted for patients with gout. [22,91].

## 7. Future Perspectives

Increasing knowledge on the inflammatory mechanisms involved in gout in response to MSU crystals should aid in the development of new therapeutic compounds in the near future. Apart from anti-cytokines such as anti-IL-1, other new therapies should be identified to target the different components of the pathways involved in gout. Recently, new plant-derived natural compounds have been studied in murine models; however, the efficacy in gouty patients need to be confirmed. The therapeutic potential role of lnc-RNAs and miRNAs represents a new field of application; however, further studies are required to confirm their capability to curb or modify the inflammatory cascade involved in gout.

## 8. Concluding Remarks

Autoinflammation-related mechanisms contribute to diseases not usually considered primarily immune-mediated, including crystal-induced arthropathies. In recent years, the concept of gout moved from a purely metabolic disease to a more global autoinflammatory disease, leading to expanded treatment options targeting specific inflammatory mechanisms. Pursuing those types of therapies may provide more safe and effective alternatives for patients in the future, since gout represents the most prevalent destructive inflammatory joint disease.

## Figures and Tables

**Table 1:** Drugs and compounds proposed for gouty treatment targeting autoinflammatory mediators.

**Anti IL-1**			
	**Dosage**	**Target**	**Reference**
*Anakinra*	100 mg daily	IL-1 receptor	[72]
*Canakinumab*	150 mg at baseline	IL-1β	[76]
*Rilonacept (trap protein)*	320 mg at baseline	Trap-fusion protein blocking both IL-α and Il-1β	[74]
*lncRNA* *(miRNA-488, miRNA-920)*	NA	IL-β	[77]
**IL-1β processing inhibitors**			
	**Dosage**	**Target**	**Reference**
*VX-765 (belnacasan)*	NA	Caspase I	[78]
*A1AT*	NA	Caspase I	[79]
*MMC-950 (CRID3)*	NA	ASC complex	[37]
**NLRP3 inhibitors**			
	**Dosage**	**Target**	**Reference**
*Glucocorticoids*	variable	NLRP3 (indirectly) NF-κB pathway	[6]
*Colchicine*	1 mg/day (followed by 0.5 mg after 30 min on day 1)	Microtubules polymerization, Chemokines, chemotaxis, NLRP3	[6]
*Dapansutrile (OLT 1177)*	100 mg/day, 300 mg/day, 1000 mg/day, or 2000 mg/day orally for 8 days	NLRP3	[80]
*Beta-hydroxybutyrate (BHB)*	NA	NLRP3, K+ channels	[81]
*Polyphenols present in food (ProcyanidinB2, Curcumin, Epigallocatechingallate)*	NA	NLRP3	[37,82,83]
*Carotenoids (Beta-carotene)* *Other compounds: Eucalyptol, Omega3 FAs, small peptides (RDP3), vitamins (riboflavin-B2)*	NA	NLRP3	[82,84,85,86,87,88]
**Receptor inhibitors**			
	**Dosage**	**Target**	**Reference**
*lncRNA (miRNA-146a)*	NA	Myd88/*TLR4*	[47]

NA: not available.

## Data Availability

Not applicable.
